# Synergistic nutrient uptake and utilization in maize varieties with different nitrogen efficiencies

**DOI:** 10.3389/fpls.2026.1867272

**Published:** 2026-07-29

**Authors:** Yanzhi Tan, Yuping Wang, Guowei Chen, Yahan Zhang, Qin Huang, Junlan Liu, Haodan Zhang, Ailin Tian, Linyu Liu, Guoqing Sun, Qiang Li, Linzheng Liao

**Affiliations:** Chongqing Key Laboratory for Germplasm Innovation of Special Aromatic Spice Plants, College of Smart Agriculture/Institute of Special Plants, Chongqing University of Arts and Sciences, Chongqing, China

**Keywords:** absorption and utilization, maize, N-efficiency, nutrient, synergistic effect

## Abstract

Numerous studies have examined nutrient accumulation and distribution in maize varieties with varying nitrogen (N) efficiencies, yet research on their coordinated nutrient uptake and utilization under field conditions remains insufficiently quantified. We studied differences in the absorption, accumulation, and utilization of N, phosphorus (P), and potassium (K) in the roots of N-efficient maize variety ZH 311 and N-inefficient variety XY 508 under four N levels from 2024 to 2025. Root dry matter, stage-specific uptake rates, nutrient absorption efficiency, apparent utilization efficiency, yield components, and grain yield were also evaluated. The absorption efficiency and accumulation of N, P, and K in the roots of ZH 311 were significantly higher than those in XY 508, with positive relationships observed among N, P, and K accumulation. The accumulation peaks of N, P, and K occurred at V12-R1, R3-R6, and V6-V12, respectively, indicating stage-specific coordination rather than completely synchronous uptake. Principal component analysis based on six nutrient-efficiency indicators showed that PC1 and PC2 explained 57.54% and 32.45% of the total variation, respectively. These results show the superior N, P, and K uptake and utilization efficiency of N-efficient varieties compared with N-inefficient ones. Yield analysis further showed that ZH 311 achieved the highest yield under N240, whereas XY 508 reached the highest yield under N360, suggesting that moderate N input can better match the nutrient-efficiency advantage of ZH 311.

## Graphical Abstract

**Figure:**
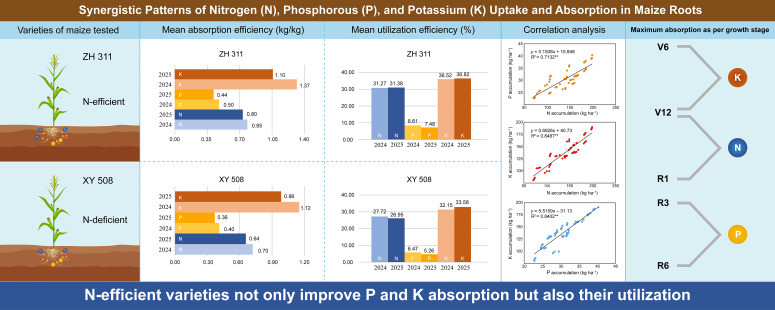

## 1 Introduction

Maize (*Zea mays* L.) is one of the most important cereal crops worldwide and plays a major role in food, feed, and industrial production (Hu and Liu, 2023; Li, 2024). As such, maize production is crucial for global food security. China is the world’s second largest producer of maize; the area of maize planted and its yield in China represent 22% and 24% of the global total, respectively, and it thus makes significant contributions to global maize production (Tian et al., 2020). Nitrogen (N) is one of the most important and widely absorbed nutrients in plants; it has significant effects on plant metabolism, physiology, material production, organ formation, and source-sink relationships, and is an important factor limiting plant yield and quality (Dong et al., 2023; Ai and Wei, 2024; Lu et al., 2009). Increasing N application is a direct and effective way to increase maize yield. To obtain high yields in China, excessive amounts of N are generally applied, which is not only a significant waste of resources, but it results in environmental problems such as the degradation of cultivated land quality and water pollution. Furthermore, it causes a decline in the utilization efficiency of N (Hou et al., 2021; Wang et al., 2012; Wang et al., 2014; Zhao and Li, 2024). Therefore, improving N-use efficiency through genotype selection and appropriate N management is an important approach for achieving high-yield and resource-efficient maize production.

N, phosphorus (P), and potassium (K) are the three most important nutrients for plant growth and development, and their absorption, accumulation, distribution, and utilization are essential for plant growth and yield (Yang et al., 2015 Ma, 2023; Zhu et al., 2019). Maize shows obvious genotypic differences between the absorption, utilization, and distribution of N, P, and K in different varieties (Chang, 2010; Li et al., 2010; Qi et al., 2013; Huang et al., 2018; Wang et al., 2009). In this respect, Li et al. (2022a) showed that N-efficient maize varieties have a stronger N absorption capacity, higher N accumulation capacity, and higher N transport capacity than N-inefficient varieties, and their N absorption efficiency, utilization efficiency, and agronomic efficiency are higher. Huang et al. (2021) found that the roots of wheat varieties with a high P efficiency were more developed than those with a low P efficiency, and P accumulation and transport before and after anthesis were higher, resulting in the higher P absorption and utilization efficiency. Owing to the interaction effect between plant nutrient elements, a change in one nutrient element will also affect the absorption and utilization of other elements by plants (Li et al., 2021; Liu et al., 2024). Song et al. (2024) found that N-efficient blueberry varieties had higher N absorption and utilization efficiencies than N-inefficient varieties, and their absorption efficiencies of P and K were also higher. In addition, the P accumulation of P-efficient cotton varieties was found to be significantly higher than that of the P-inefficient varieties, and the accumulation of N and K was significantly higher than that of the P-inefficient varieties (Luo et al., 2017). These findings suggest that nutrient efficiency is not an isolated single-element trait, but may involve coordinated acquisition and utilization of multiple nutrients.

At the molecular level, N uptake and long-distance transport are mainly mediated by nitrate and ammonium transport systems, including NPF/NRT1, NRT2, and AMT family members; P uptake is primarily controlled by phosphate transporter families such as PHT1; and K acquisition depends on K+ channels and transporters, including AKT and HAK/KUP/KT systems (Lu and Yang, 2006). These transporter systems may interact with root growth, carbon allocation, and rhizosphere nutrient processes, providing a mechanistic basis for N-P-K coordination (Krouk et al., 2010; Bouain et al., 2019; Massucato et al., 2022; Zhang et al., 2021; Sun et al., 2023).

Many studies have been conducted on the differences in material production, N accumulation, distribution, absorption, and utilization in maize varieties with different N efficiencies. However, field evidence directly comparing root-stage N, P, and K uptake rates and nutrient-use efficiencies between maize varieties with contrasting N efficiency remains limited, and the contribution of such coordination to yield formation has not been sufficiently clarified. Therefore, to provide a theoretical basis and technical support for the synergistic absorption of plant nutrients, maize varieties with different N efficiencies screened in the early stage were studied under field conditions to analyze differences in N, P, and K accumulation, root absorption rates, and N, P, and K absorption and utilization efficiencies. This study further aimed to clarify whether an N-efficient maize variety also exhibits stronger coordinated P and K uptake and utilization under low to moderate N supply.

## 2 Materials and methods

### 2.1 Experimental materials

N-efficient Zhenghong 311 (ZH 311) and N-inefficient Xianyu 508 (XY 508) varieties were studied; these are the main maize varieties in Southwest China, and they have a growth period of approximately 120 days. The selection of ZH 311 and XY 508 was based on previous low-N tolerance screening and subsequent field validation studies from Qiang Li’s research group and collaborators. ZH 311 was identified as a low-N-tolerant cultivar, whereas XY 508 was classified as low-N-sensitive; subsequent studies used them as representative varieties with contrasting N efficiencies (Li et al., 2014; Du et al., 2020; Li et al., 2022a; Liu et al., 2025; Bao et al., 2025).

### 2.2 Overview of experimental site

**Figure 1:**
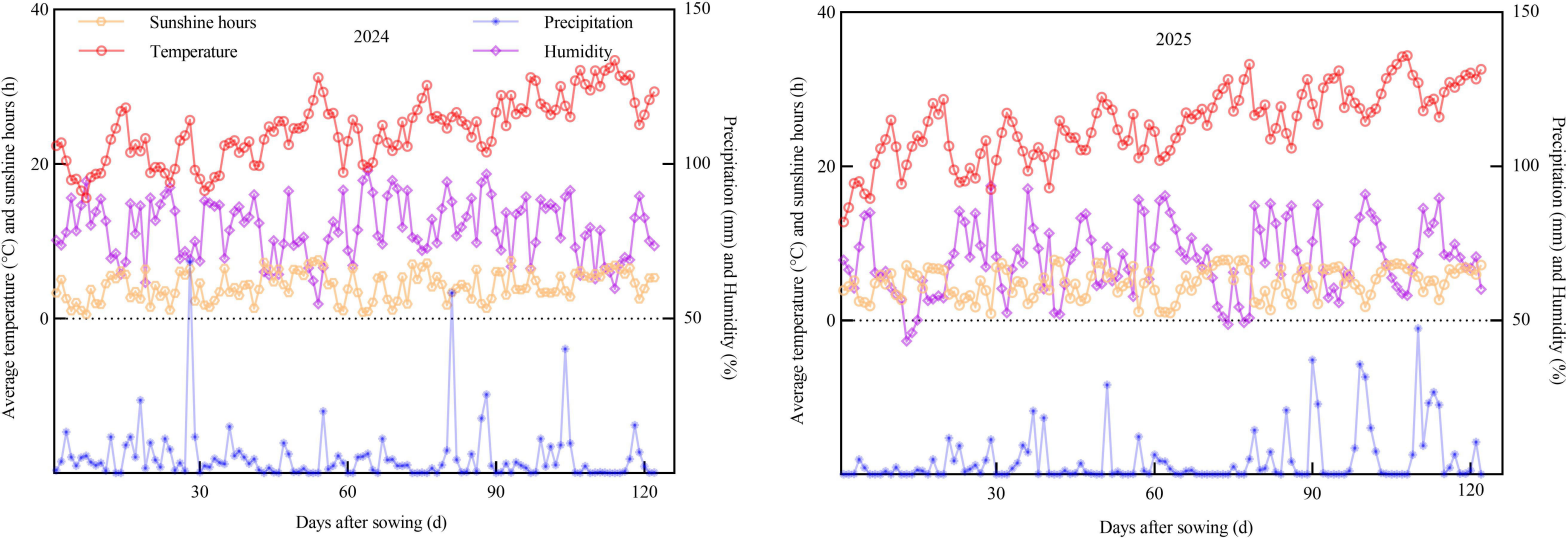
Meteorological factors during maize growth.

The experiment was conducted in Yongchuan, Chongqing (29° 21’N, 105° 54’E) from 2024 to 2025. The climate at the site is subtropical monsoon, and the meteorological factors affecting maize growth are shown in **Figure 1**. The test soil is purple (Orthic Entisols). The basic nutrients at a soil depth of 0–30 cm in 2024 (2025) were as follows: pH 7.92 (7.63), total N 1.63 g kg^−1^ (1.55 g kg^−1^), total P 0.62 g kg^−1^ (0.49 g kg^−1^), total K 11.55 g kg^−1^ (9.74 g kg^−1^), available N 48.72 mg kg^−1^ (39.77 mg kg^−1^), available P 2.68 mg kg^−1^ (2.37 mg kg^−1^). available K 145.21 mg kg^−1^ (131.19 mg kg^−1^), organic matter 16.14 g kg^−1^ (13.97 g kg^−1^).

### 2.3 Experimental design

A two-factor randomized block design was employed that included two factors: the maize variety (N-efficient variety ZH 311 and N-inefficient variety XY 508) and the level of N fertilizer (N1:0 kg ha^−1^, N2:120 kg ha^−1^, N3:240 kg ha^−1^, N4:360 kg ha^−1^). Each treatment was repeated three times, with a total of 24 plots, plots 5 m × 8 m (40 m2). Maize was planted in a wide-narrow row (1.4 m + 0.6 m) with a density of 52500 plants ha−1. Urea was used as the N fertilizer; 50% of the total N was applied as basal fertilizer before sowing, and the remaining 50% was topdressed at the big-bell stage (V12). Phosphate (600 kg ha^−1^ of superphosphate) and K fertilizer (150 kg ha^−1^ of K chloride) were applied once simultaneously as base fertilizers. Pest and weed control was performed according to a local high-yield cultivation demonstration (Zhu and Shan, 2020).

### 2.4 Sampling and measurement

At the V6 (sixth leaf), V12 (twelfth leaf/big-bell), R1 (silking), R3 (milk), and R6 (physiological maturity) stages, five continuous plants with similar growth vigor were collected from each plot. Root sampling was conducted by whole-plant excavation. The whole root system was excavated to a depth of 0–80 cm with a radius of 12.5 cm around the plant, corresponding to the 25 cm plant spacing. The excavated root system was wrapped in a nylon mesh bag and carefully washed with water to remove adhering soil and reduce fine-root loss. Roots, stem sheaths, leaves, and ears were then separated. After deactivation at 105 °C for 30 min, samples were dried to constant weight at 80 °C, weighed, crushed, and passed through a 60-mesh sieve. Samples were digested using the H_2_O_2_-H_2_SO_4_ wet ashing method. N, P, and K contents were determined using the Kjeldahl method (Li et al., 2023), vanadium molybdenum yellow colorimetric method (Liu et al., 2021), and flame photometry (Tan et al., 2016), respectively.

### 2.5 Data statistics and analysis

Excel 2019 was used for data collection. The SPSS software package (version 20.0) was employed to analyze the data using the least significant difference method to test for significant differences (significance level p<0.05) between N levels and varieties. GraphPad Prism software (version 5.0) was used to conduct the statistical analyses. Data for each treatment are presented as means ± standard deviation (SD, n = 3). Pearson correlation and linear regression were used to evaluate the relationships among N, P, and K accumulation. Principal component analysis (PCA) was conducted after standardizing six nutrient-efficiency indicators: N, P, and K absorption efficiencies and N, P, and K apparent utilization efficiencies.

To improve reproducibility, nutrient accumulation (NA) was calculated as NA = DM × C/1000, where DM is dry matter accumulation and C is nutrient concentration. Stage nutrient accumulation (SNA) was calculated as the difference in nutrient accumulation between two adjacent growth stages. Root nutrient uptake rate (RUR) was calculated as SNA/[D × (RDM1 + RDM2)/2], where D is the number of days between two adjacent stages and RDM1 and RDM2 are root dry matter at the two stages. Apparent utilization efficiency (AUE) was calculated as AUE (%) = (UN − U0)/F × 100, where UN is nutrient uptake under fertilization, U0 is nutrient uptake in the control, and F is the applied fertilizer amount. Grain yield was converted from kg mu−1 to t ha−1 using a conversion coefficient of 0.015.

## 3 Results

### 3.1 Difference in root dry matter accumulation of maize varieties with different N efficiency

The dry matter accumulation of maize roots increased gradually with the growth process, and that of the two varieties was the highest at the mature stage in both years (**Table 1**). There was no significant difference in the root dry matter accumulation between the two varieties in V6, V12, R1, and R3, whereas accumulation was significantly higher in ZH 311 than XY 508 in R6 (*p* < 0.01). On average, the root dry matter of ZH 311 was 9.56% higher than that of XY 508 at R6 in 2024 and 15.79% higher than XY 508 in 2025, indicating that ZH 311 maintained a higher root dry matter and delayed root senescence at a later stage of growth than XY 508. The root dry matter accumulation in the two varieties increased significantly, but there were significant differences between the varieties. On average, compared with the N0 treatment, N application increased the root dry matter accumulation of ZH 311 by 30.33%, 35.42%, 30.95%, 26.16%, and 18.02% at the V6, V12, R1, R3, and R6 stages, respectively, in 2024 and by 22.27%, 28.34%, 11.95%, 13.68%, and 28.39% in 2025. Additionally, compared with the N0 treatment, the root dry matter increased of ZH 311 by an average of 18.99%, 32.91%, and 25.32% across growth stages under N120, N240, and N360 treatments, respectively, in 2024, and by 16.25%, 28.75%, and 17.50% in 2025. For XY 508, the increases were 26.75%, 48.89%, 32.41%, 27.65%, and 24.22% in 2024, and 28.28%, 41.87%, 17.98%, 20.49%, and 17.51% in 2025, respectively. Compared with the N0 treatment, the root dry matter increased of XY 508 by an average of 21.62%, 29.73%, and 40.54% across growth stages under N120, N240, and N360 treatments, respectively, in 2024, and by 13.92%, 22.78%, and 24.05% in 2025. Overall, XY 508 exhibited significantly greater increases in root dry matter accumulation across growth stages than ZH 311 (except for V6 in 2024 and R6 in 2025), indicating that N application more effectively enhanced root dry matter accumulation in the N-inefficient variety XY 508.

**Table 1:** Difference of root dry matter accumulation in different stages (t ha^−1^).

Varieties	Nitrogen rate	V6		V12		R1		R3		R6		Mean	
		2024	2025	2024	2025	2024	2025	2024	2025	2024	2025	2024	2025
ZH 311	N 0	0.31 ± 0.05 c	0.34 ± 0.00 e	0.62 ± 0.02 d	0.66 ± 0.02 e	0.77 ± 0.04 d	0.92 ± 0.02 c	0.93 ± 0.06 d	1.00 ± 0.03 d	1.31 ± 0.03 bcd	1.09 ± 0.03 cd	0.79 f	0.80 e
	N 120	0.37 ± 0.01 b	0.42 ± 0.03 c	0.79 ± 0.06 c	0.78 ± 0.04 d	0.93 ± 0.04 c	1.06 ± 0.04 a	1.11 ± 0.02 c	1.12 ± 0.04 c	1.51 ± 0.12 ab	1.30 ± 0.09 b	0.94 de	0.93 d
	N 240	0.42 ± 0.04 a	0.47 ± 0.01 a	0.89 ± 0.01 ab	0.89 ± 0.02 bc	1.07 ± 0.02 a	1.09 ± 0.01 a	1.21 ± 0.03 a	1.16 ± 0.03 abc	1.65 ± 0.25 a	1.53 ± 0.06 a	1.05 a	1.03 a
	N 360	0.41 ± 0.01 ab	0.38 ± 0.01 d	0.86 ± 0.01 bc	0.86 ± 0.01 c	1.02 ± 0.02 ab	0.96 ± 0.09 bc	1.19 ± 0.04 ab	1.12 ± 0.03 bc	1.50 ± 0.03 ab	1.36 ± 0.16 b	0.99 bc	0.94 cd
	Mean	**0.37 A**	**0.40 A**	**0.79 A**	**0.80 A**	**0.94 A**	**1.01 A**	**1.11 A**	**1.10 A**	**1.49 A**	**1.32 A**	**0.94 A**	**0.92 A**
XY 508	N 0	0.31 ± 0.02 c	0.33 ± 0.01 e	0.59 ± 0.01 d	0.62 ± 0.02 e	0.74 ± 0.06 d	0.92 ± 0.02 c	0.90 ± 0.03 d	0.97 ± 0.02 d	1.15 ± 0.05 d	1.01 ± 0.03 d	0.74 g	0.79 e
	N 120	0.37 ± 0.03 ab	0.39 ± 0.01 d	0.78 ± 0.05 c	0.81 ± 0.02 d	0.97 ± 0.08 bc	1.07 ± 0.10 a	1.09 ± 0.03 c	1.11 ± 0.04 c	1.28 ± 0.16 cd	1.11 ± 0.13 cd	0.90 e	0.90 d
	N 240	0.39 ± 0.03 ab	0.44 ± 0.02 b	0.87 ± 0.09 b	0.91 ± 0.01 ab	0.95 ± 0.03 bc	1.08 ± 0.06 a	1.14 ± 0.03 bc	1.18 ± 0.03 ab	1.46 ± 0.07 abc	1.25 ± 0.06 bc	0.96 cd	0.97 bc
	N 360	0.40 ± 0.02 ab	0.45 ± 0.01 ab	0.98 ± 0.01 a	0.93 ± 0.02 a	1.00 ± 0.02 ab	1.10 ± 0.09 a	1.22 ± 0.02 a	1.21 ± 0.04 a	1.56 ± 0.03 a	1.21 ± 0.09 bc	1.04 ab	0.98 b
	Mean	**0.37 A**	**0.40 A**	**0.80 A**	**0.82 A**	**0.92 A**	**1.04 A**	**1.09 A**	**1.12 A**	**1.36 B**	**1.14 B**	**0.91 A**	**0.91 A**
*F* value	Years/Y	22.10**		0.65^ns^		36.46**		0.76^ns^		42.69**		2.62^ns^	
	Variety/V	0.11^ns^		2.88^ns^		0.12^ns^		0.00^ns^		25.36**		13.70**	
	Nitrogen/N	53.22**		162.07**		36.25**		120.13**		23.11**		208.21**	
	Y × V	0.80^ns^		0.53^ns^		4.79*		2.90^ns^		0.58^ns^		1.62^ns^	
	Y × N	1.51^ns^		1.15^ns^		4.61**		4.92**		0.32^ns^		5.33**	
	V× N	4.14*		8.85**		3.17*		4.10*		2.08^ns^		11.49**	
	Y × V× N	3.49*		1.63^ns^		1.70^ns^		1.18**		1.31^ns^		0.33^ns^	

Values are means ± SD (n = 3). Different lowercase letters in the same column represent a significant difference (*p* < 0.05); ** indicates the difference was extremely significant at the 1% level (*p* < 0.01); * indicates the difference was significant at the 5% level (*p* < 0.05); ns indicates that the difference or relationship being tested is not statistically significant.

The bolded parts are all "Mean", and in the chart, they represent the average values of different varieties and different years (N0, N120, N240, N360) of data.

### 3.2 Differences in N, P, and K accumulation in maize varieties with different N efficiency

The effects of the variety and N application on N accumulation in maize were significant (**Table 2**). N accumulation in ZH 311 in each period was significantly higher than that in XY 508 (*p* < 0.01). The average N levels were 19.16%, 19.06%, 18.25%, 18.60%, and 22.89% higher in the V6, V12, R1, R3, and R6 periods in 2024, and 10.42%, 13.29%, 19.63%, 20.04%, and 24.20% higher in 2025, respectively, indicating that ZH 311 had a stronger root N absorption capacity than XY 508. Thus, its N accumulation in each period was significantly higher than that of XY 508. N application significantly increased N accumulation in maize during each period, but there were obvious differences among the varieties. Compared with the N0 treatment, the N accumulation of ZH 311 in the V6, V12, R1, R3, and R6 stages increased by 50.54%, 52.15%, 54.26%, 55.52%, and 63.40%, respectively, in 2024, and by 66.76%, 66.83%, 70.55%, 67.36%, and 62.61%, respectively, in 2025. Additionally, compared with the N0 treatment, the N accumulation of ZH 311 by an average of 33.73%, 76.07%, and 60.78% across growth stages under N120, N240, and N360 treatments, respectively, in 2024, and by 44.66%, 86.42%, and 68.00% in 2025. In 2024, XY 508 increased by 106.62%, 97.57%, 90.20%, 76.82% and 77.75% respectively; 2025 increased by 85.07%, 72.02%, 77.66%, 84.11% and 85.94%, respectively. Compared with the N0 treatment, the N accumulation of XY 508 by an average of 56.66%, 92.41%, and 104.05% across growth stages under N120, N240, and N360 treatments, respectively, in 2024, and by 49.73%, 87.77%, and 107.27% in 2025. The increase in N accumulation in XY 508 was significantly higher than in ZH 311, indicating that increasing N application was more conducive to increasing N accumulation in XY 508.

**Table 2:** Difference of N accumulation in different stages (kg ha^−1^).

Varieties	Nitrogen rate	V6		V12		R1		R3		R6		Mean	
		2024	2025	2024	2025	2024	2025	2024	2025	2024	2025	2024	2025
ZH 311	N 0	23.58 ± 1.06 f	22.11 ± 1.06 f	48.37 ± 0.95 f	41.23 ± 0.16 f	76.44 ± 1.53 g	63.52 ± 1.78 f	95.12 ± 1.56 g	83.08 ± 1.92 g	107.50 ± 1.18 f	100.51 ± 4.85 f	70.20 g	62.09 f
	N 120	31.38 ± 1.92 d	32.86 ± 0.79 d	64.14 ± 1.36 d	60.49 ± 1.31 d	100.14 ± 0.74 e	95.14 ± 2.41 d	126.37 ± 1.13 e	119.05 ± 1.81 e	147.40 ± 2.69 d	141.58 ± 2.49 d	93.88 e	89.82 d
	N 240	38.90 ± 2.01 a	41.62 ± 0.43 a	82.51 ± 3.47 a	76.92 ± 1.22 a	132.38 ± 2.21 a	120.10 ± 1.52 a	165.86 ± 3.01 a	156.40 ± 1.80 a	198.35 ± 1.20 a	183.72 ± 2.28 a	123.60 a	115.75 a
	N 360	36.23 ± 0.75 b	36.13 ± 0.20 b	74.13 ± 1.83 b	68.93 ± 0.24 b	121.21 ± 2.18 b	109.76 ± 1.96 b	151.56 ± 2.51 b	141.68 ± 2.85 b	181.24 ± 1.62 b	165.04 ± 3.52 b	112.87 b	104.31 b
	Mean	**32.52 A**	**33.18 A**	**67.29 A**	**61.89 A**	**107.54 A**	**97.13 A**	**134.73 A**	**125.05 A**	**158.62 A**	**147.71 A**	**100.14 A**	**92.99 A**
XY 508	N 0	15.17 ± 1.24 g	18.34 ± 0.27 g	32.64 ± 1.82 g	35.47 ± 0.53 g	54.24 ± 4.51 h	51.31 ± 1.36 g	72.07 ± 4.12 h	63.88 ± 1.54 h	81.53 ± 3.65 g	72.32 ± 1.60 g	51.13 h	48.26 g
	N 120	28.08 ± 0.72 e	30.20 ± 0.37 e	53.22 ± 1.52 e	53.01 ± 0.66 e	87.02 ± 8.09 f	77.55 ± 1.20 e	109.16 ± 7.12 f	93.69 ± 2.01 f	123.01 ± 5.01 e	106.88 ± 1.47 e	80.10 f	72.26 e
	N 240	31.88 ± 0.77 d	34.78 ± 1.17 c	67.79 ± 1.73 c	62.82 ± 0.07 c	108.26 ± 2.81 d	94.12 ± 2.62 d	133.20 ± 2.72 d	123.00 ± 1.81 d	150.76 ± 2.71 d	138.37 ± 3.58 d	98.38 d	90.62 d
	N 360	34.05 ± 1.09 c	36.87 ± 0.92 b	72.42 ± 1.57 b	67.23 ± 2.20 b	114.25 ± 2.45 c	101.78 ± 1.15 c	139.96 ± 2.78 c	136.12 ± 2.29 c	161.00 ± 3.83 c	158.16 ± 2.07 c	104.33 c	100.03 c
	Mean	**27.29 B**	**30.05 B**	**56.52 B**	**54.63 B**	**90.94 B**	**81.19 B**	**113.60 B**	**104.17 B**	**129.07 B**	**118.93 B**	**83.49 B**	**77.79 B**
*F* value	Years/Y	43.06**		80.86**		165.70**		163.99**		158.88**		253.50**	
	Variety/V	259.50**		495.97**		431.47**		792.78**		1219.24**		1561.35**	
	Nitrogen/N	900.71**		1422.35**		969.63**		1728.16**		1920.25**		3253.20**	
	Y × V	16.31**		18.81**		0.18^ns^		0.03^ns^		0.21^ns^		3.27^ns^	
	Y × N	2.58^ns^		5.21**		3.55*		1.66^ns^		1.91^ns^		1.54^ns^	
	V × N	30.41**		43.51**		21.23**		44.79**		65.37**		90.52**	
	Y × V × N	4.00*		7.94**		4.16*		4.42*		8.73**		6.60**	

The bolded parts are all "Mean", and in the chart, they represent the average values of different varieties and different years (N0, N120, N240, N360) of data.

Values are means ± SD (n = 3). Different lowercase letters in the same column represent a significant difference (p < 0.05); ** indicates the difference was extremely significant at the 1% level (p < 0.01); * indicates the difference was significant at the 5% level (p < 0.05); ns indicates that the difference or relationship being tested is not statistically significant.

The variety type and N application had significant effects on P accumulation in maize at different stages (**Table 3**). The P accumulation in ZH 311 in each period was significantly higher than that in XY 508 (*p* < 0.01). The average N levels of V6, V12, R1, R3, and R6 in 2024 were 12.17%, 17.03%, 24.33%, 20.73%, and 21.55% higher than those of XY 508, respectively, and 8.04%, 28.27%, 17.70%, 15.63%, and 21.89% higher in 2025, respectively, indicating that ZH 311 had a stronger root P absorption capacity than XY 508. Therefore, its P accumulation in each period was significantly higher than that of XY 508. N application significantly increased P accumulation in maize during each period; however, there were significant differences among the varieties. Compared with the N0 treatment, the P accumulation of ZH 311 in V6, V12, R1, R3, and R6 increased by 60.05%, 30.48%, 10.11%, 17.46%, and 19.78% respectively in 2024, and increased by 47.22%, 39.56%, 22.68%, 16.14%, and 13.52%, respectively, in 2025. Additionally, compared with the N0 treatment, the P accumulation of ZH 311 by an average of 12.75%, 26.40%, and 20.99% across growth stages under N120, N240, and N360 treatments, respectively, in 2024, and by 13.85%, 27.84%, and 18.75% in 2025. In 2024, XY 508 increased by 58.43%, 38.81%, 22.78%, 19.93% and 16.81% respectively; in 2025, it increased by 47.24%, 43.22%, 25.16%, 20.80% and 16.88% respectively. Compared with the N0 treatment, the P accumulation of XY 508 by an average of 14.72%, 24.40%, and 29.04% across growth stages under N120, N240, and N360 treatments, respectively, in 2024, and by 15.39%, 24.42%, and 31.56% in 2025. There was no significant difference in the increase in P accumulation between the two varieties in the early (V6) and late growth stages (R6), whereas the increase in P accumulation in the middle growth stages (V12, R1, and R3) of XY 508 was significantly higher than that of ZH 311, indicating that N application was more conducive to improving P accumulation in the N-inefficient variety XY 508 during the middle growth stage.

**Table 3:** Difference of P accumulation in different stages (kg ha^−1^).

Varieties	Nitrogen rate	V6		V12		R1		R3		R6		Mean	
		2024	2025	2024	2025	2024	2025	2024	2025	2024	2025	2024	2025
ZH 311	N 0	3.18 ± 0.10 e	3.58 ± 0.11 f	7.50 ± 0.24 e	7.51 ± 0.16 g	13.83 ± 0.27 d	10.69 ± 0.20 e	21.78 ± 0.51 de	20.06 ± 0.28 d	31.34 ± 0.76 d	28.56 ± 0.82 d	15.53 e	14.08 f
	N 120	4.56 ± 0.28 c	4.93 ± 0.19 cd	8.70 ± 0.17 d	9.88 ± 0.29 c	14.61 ± 0.23 c	12.32 ± 0.27 c	24.31 ± 0.21 c	22.05 ± 0.51 c	35.39 ± 1.14 c	30.97 ± 0.49 c	17.51 c	16.03 c
	N 240	5.50 ± 0.29 a	5.83 ± 0.15 a	10.52 ± 0.08 a	11.24 ± 0.10 a	15.78 ± 0.16 a	13.83 ± 0.40 a	26.82 ± 0.21 a	24.77 ± 0.39 a	39.52 ± 0.73 a	34.32 ± 0.51 a	19.63 a	18.00 a
	N 360	5.20 ± 0.14 b	5.04 ± 0.11 c	10.14 ± 0.12 b	10.32 ± 0.03 b	15.31 ± 0.14 b	13.18 ± 0.20 b	25.62 ± 0.36 b	23.07 ± 0.33 b	37.69 ± 0.36 b	31.99 ± 0.31 b	18.79 b	16.72 b
	Mean	**4.61 A**	**4.84 A**	**9.21 A**	**10.48 A**	**15.23 A**	**12.50 A**	**24.64 A**	**22.49 A**	**35.98 A**	**31.46 A**	**17.87 A**	**16.21 A**
XY 508	N 0	2.86 ± 0.15 f	3.31 ± 0.03 g	6.10 ± 0.09 f	6.17 ± 0.17 h	10.46 ± 0.36 h	8.94 ± 0.33 f	17.75 ± 0.48 g	16.82 ± 0.42 f	25.34 ± 0.61 g	22.91 ± 0.14 g	12.50 g	11.63 h
	N 120	3.97 ± 0.13 d	4.50 ± 0.05 e	7.52 ± 0.15 e	8.19 ± 0.14 f	12.11 ± 0.19 g	10.63 ± 0.25 e	20.44 ± 0.07 f	18.88 ± 0.34 e	27.67 ± 0.72 f	24.89 ± 0.21 f	14.34 f	13.42 g
	N 240	4.79 ± 0.17 c	5.27 ± 0.06 b	8.84 ± 0.09 cd	8.89 ± 0.20 e	13.01 ± 0.12 f	11.23 ± 0.19 d	21.33 ± 0.19 e	20.33 ± 0.29 d	29.79 ± 0.72 e	26.66 ± 0.26 e	15.55 e	14.47 e
	N 360	4.81 ± 0.09 c	4.84 ± 0.02 d	9.02 ± 0.22 c	9.43 ± 0.10 d	13.40 ± 0.14 e	11.70 ± 0.07 d	22.10 ± 0.32 d	21.76 ± 0.37 c	31.33 ± 0.75 d	28.78 ± 0.26 d	16.13 d	15.30 d
	Mean	**4.11 B**	**4.48 B**	**7.87 B**	**8.17 B**	**12.25 B**	**10.62 B**	**20.41 B**	**19.45 B**	**29.60 B**	**25.81 B**	**14.63 B**	**13.71 B**
*F* value	Years/Y	51.92**		75.60**		918.63**		228.52**		401.02**		691.65**	
	Variety/V	107.96**		943.25**		1169.37**		1251.52**		1311.68**		3407.94**	
	Nitrogen/N	485.023**		887.91**		308.64**		362.11**		210.32**		1075.58**	
	Y × V	2.80^ns^		5.48*		32.90**		33.62**		24.62**		55.64**	
	Y × N	8.74**		15.54**		2.86^ns^		1.53^ns^		4.05*		1.88^ns^	
	V × N	3.95*		19.42**		11.81**		25.89**		21.25**		54.11**	
	Y × V × N	0.12^ns^		5.37**		5.78**		2.88^ns^		2.59^ns^		2.97*	

The bolded parts are all "Mean", and in the chart, they represent the average values of different varieties and different years (N0, N120, N240, N360) of data.

Values are means ± SD (n = 3). Different lowercase letters in the same column represent a significant difference (p < 0.05); ** indicates the difference was extremely significant at the 1% level (p < 0.01); * indicates the difference was significant at the 5% level (p < 0.05); ns indicates that the difference or relationship being tested is not statistically significant.

The variety and N application significantly affected K accumulation in the maize at each stage (**Table 4**). K accumulation in ZH 311 in each period was significantly higher than that in XY 508 (*p* < 0.01). The average N levels of V6, V12, R1, R3, and R6 in 2024 were 21.58%, 11.67%, 15.05%, 18.60%, and 22.55% higher than those of XY 508, respectively, and 9.25%, 10.00%, 11.53%, 12.43%, and 14.47% higher in 2025, respectively, indicating that ZH 311 had a stronger K absorption capacity than XY 508. Therefore, the amount of K accumulated in each period was significantly higher than that of XY 508. N application significantly increased K accumulation in maize during each period; however, there were significant differences among the varieties. Compared with the N0 treatment, the amount of K accumulated by ZH 311 increased by 38.49%, 23.21%, 24.96%, 28.68%, and 31.81% at V6, V12, R1, R3, and R6 in 2024, respectively, and by 39.28%, 39.22%, 40.46%, 39.27%, and 45.49%, respectively, in 2025. Additionally, compared with the N0 treatment, the K accumulation of ZH 311 by an average of 19.21%, 36.59%, and 30.07% across growth stages under N120, N240, and N360 treatments, respectively, in 2024, and by 29.81%, 57.06%, and 37.10% in 2025. In 2024, that of XY 508 increased by 43.16%, 29.46%, 30.56%, 32.25% and 35.40% respectively, and by 43.67%, 40.42%, 44.84%, 45.40% and 50.90% respectively in 2025. Compared with the N0 treatment, the K accumulation of XY 508 by an average of 22.66%, 34.79%, and 41.70% across growth stages under N120, N240, and N360 treatments, respectively, in 2024, and by 30.93%, 48.57%, and 58.23% in 2025. The increase in K accumulation in XY 508 was significantly higher than that in ZH 311 in each period of the two years, indicating that the increase in N fertilizer was more conducive to an increase in K accumulation in XY 508.

**Table 4:** Difference of K accumulation in different stages (kg ha^−1^).

Varieties	Nitrogen rate	V6		V12		R1		R3		R6		Mean	
		2024	2025	2024	2025	2024	2025	2024	2025	2024	2025	2024	2025
ZH 311	N 0	27.89 ± 0.17 c	25.83 ± 0.79 e	69.38 ± 1.41 e	53.38 ± 2.55 d	90.02 ± 2.10 e	69.55 ± 1.58 e	114.23 ± 1.41 e	88.22 ± 1.35 e	133.13 ± 2.44 f	98.60 ± 1.98 e	86.93 f	67.12 e
	N 120	34.91 ± 2.14 b	33.07 ± 0.62 c	80.36 ± 2.25 c	68.30 ± 2.40 b	104.61 ± 1.06 cc	89.89 ± 0.23 c	136.00 ± 1.03 c	113.48 ± 2.34 c	162.29 ± 2.38 c	130.91 ± 0.49 c	103.63 c	87.13 c
	N 240	41.24 ± 2.38 a	40.43 ± 1.03 a	89.39 ± 1.03 a	82.97 ± 1.97 a	118.66 ± 1.46 a	108.58 ± 1.52 a	156.58 ± 4.55 a	135.99 ± 1.31 a	187.83 ± 2.39 a	159.14 ± 2.30 a	118.74 a	105.42 a
	N 360	39.75 ± 0.69 a	34.41 ± 0.54 bc	86.72 ± 1.36 b	71.68 ± 1.01 b	114.18 ± 2.38 b	94.61 ± 1.54 b	148.39 ± 1.96 b	119.13 ± 1.32 b	176.32 ± 1.93 b	140.30 ± 2.98 b	113.07 b	92.02 b
	Mean	**35.95 A**	**33.43 A**	**81.46 A**	**69.08 A**	**106.87 A**	**90.66 A**	**138.80 A**	**114.21 A**	**164.90 A**	**132.24 A**	**105.59 A**	**87.92 A**
XY 508	N 0	22.34 ± 1.22 d	23.05 ± 0.18 f	58.92 ± 0.69 f	48.19 ± 1.89 e	75.56 ± 1.93 f	60.83 ± 2.52 f	94.23 ± 1.65 f	75.77 ± 2.78 f	106.33 ± 0.86 g	83.61 ± 2.63 f	71.48 g	58.29 f
	N 120	29.57 ± 1.40 c	30.03 ± 1.57 d	71.33 ± 1.66 e	62.12 ± 2.11 c	91.24 ± 0.78 e	78.67 ± 3.04 d	115.07 ± 2.37 e	98.67 ± 1.15 d	131.20 ± 1.37 f	112.09 ± 2.81 d	87.68 f	76.32 d
	N 240	32.78 ± 1.21 b	34.24 ± 1.07 bc	77.48 ± 1.23 d	69.25 ± 1.73 b	99.60 ± 1.74 d	89.30 ± 0.74 c	126.12 ± 1.25 d	111.96 ± 0.18	145.76 ± 2.53 e	128.23 ± 2.47 c	96.35 e	86.60 c
	N 360	33.59 ± 1.01 b	35.09 ± 0.55 b	80.05 ± 0.40 c	71.64 ± 1.44 b	105.15 ± 0.53 c	96.35 ± 3.17 b	132.68 ± 1.06 c	119.90 ± 1.77 bc	154.96 ± 2.65 d	138.17 ± 1.38 b	101.29 d	92.23 b
	Mean	**29.57 B**	**30.60 B**	**71.95 B**	**62.80 B**	**92.89 B**	**81.29 B**	**117.03 B**	**101.58 B**	**134.56 B**	**115.52 B**	**89.20 B**	**78.36 B**
*F* value	Years/Y	4.49*		485.36**		745.69**		1377.08**		1658.65**		1959.21**	
	Variety/V	174.13**		261.42**		525.56**		1016.59**		1373.86**		1624.45**	
	Nitrogen/N	252.38**		422.28**		743.39**		1126.30**		1305.08**		1915.24**	
	Y × V	25.84**		10.94**		20.54**		71.81**		115.17**		112.47**	
	Y × N	1.76^ns^		6.83**		8.89**		3.78*		3.29*		10.59**	
	V × N	7.69**		15.68**		38.91**		56.41**		65.40**		88.07**	
	Y × V × N	2.50^ns^		3.60*		5.51**		5.19**		2.20^ns^		8.07**	

The bolded parts are all "Mean", and in the chart, they represent the average values of different varieties and different years (N0, N120, N240, N360) of data.

Values are means ± SD (n = 3). Different lowercase letters in the same column represent a significant difference (p < 0.05); ** indicates the difference was extremely significant at the 1% level (p < 0.01); * indicates the difference was significant at the 5% level (p < 0.05); ns indicates that the difference or relationship being tested is not statistically significant.

### 3.3 Differences in N, P and K accumulation and absorption rate of different N efficiency maize varieties at different stages

**Figure 2:**
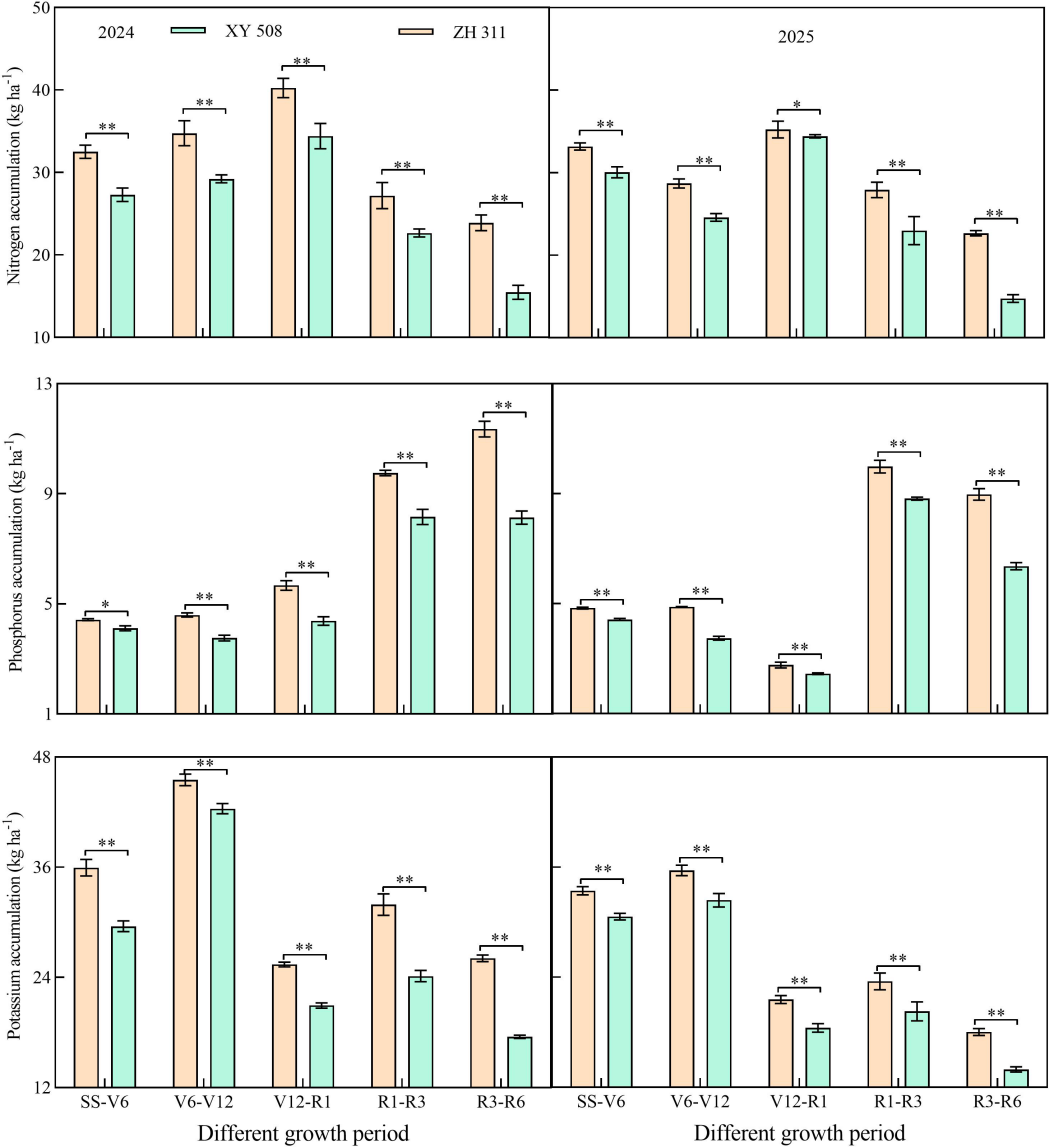
Differences of N, P, K accumulation during different growth period.

N accumulation in maize occurs mainly during the early growth stages. N accumulation in SS-V6, V6-V12 and V12-R1 was significantly higher than that in R1-R3 and R3-R6, and that of the two varieties was the highest in the V12-R1 stage in both years (**Figure 2**, **Supplementary Table 1**). P accumulation in maize is mainly concentrated during the late growth stages. P accumulation in R1-R3 and R3-R6 was significantly higher than in SS-V6, V6-V12 and V12-R1. That of the two varieties was the highest in R3-R6 in 2024, and the highest in R1-R3 in 2025. K accumulation in maize was similar to N accumulation. K accumulation in SS-V6 and V6-V12 was significantly higher than that in V12-R1, R1-R3 and R3-R6, but the accumulation peak appeared earlier. That of the two varieties was the highest during the V6-V12 stage in both years.

The accumulation of N, P, and K at different stages in maize varieties with different N efficiencies differed significantly (*p* < 0.05). The accumulation of N, P, and K at each stage in ZH 311 was significantly higher than that in XY 508. In 2024, the accumulation of N in the SS-V6, V6-V12, V12-R1, R1-R3, and R3-R6 stages increased by 19.16%, 15.93%, 14.48%, 16.66%, and 35.24%, respectively. P accumulation increased by 8.00%, 22.37%, 29.54%, 19.52%, and 39.64%, respectively. The amount of K accumulated increased by 21.56%, 7.41%, 21.33%, 32.29%, and 48.81%, respectively. In 2025, the amount of N accumulated increased by 10.43%, 16.78%, 2.36%, 21.49%, and 53.52%, respectively; the amount of P accumulated increased by 9.36%, 30.77%, 12.77%, 13.14%, and 41.16%, respectively; and the amount of K accumulated increased by 9.25%, 10.00%, 16.68%, 16.09%, and 29.26%, respectively. The increased N, P, and K accumulated in the R3-R6 stage was significantly higher than that in other stages; however, the amounts of N, P, and K accumulated in each growth stage within the N-efficient variety ZH 311 were higher than those of XY 508, but this was more obvious in the later growth stage.

**Figure 3:**
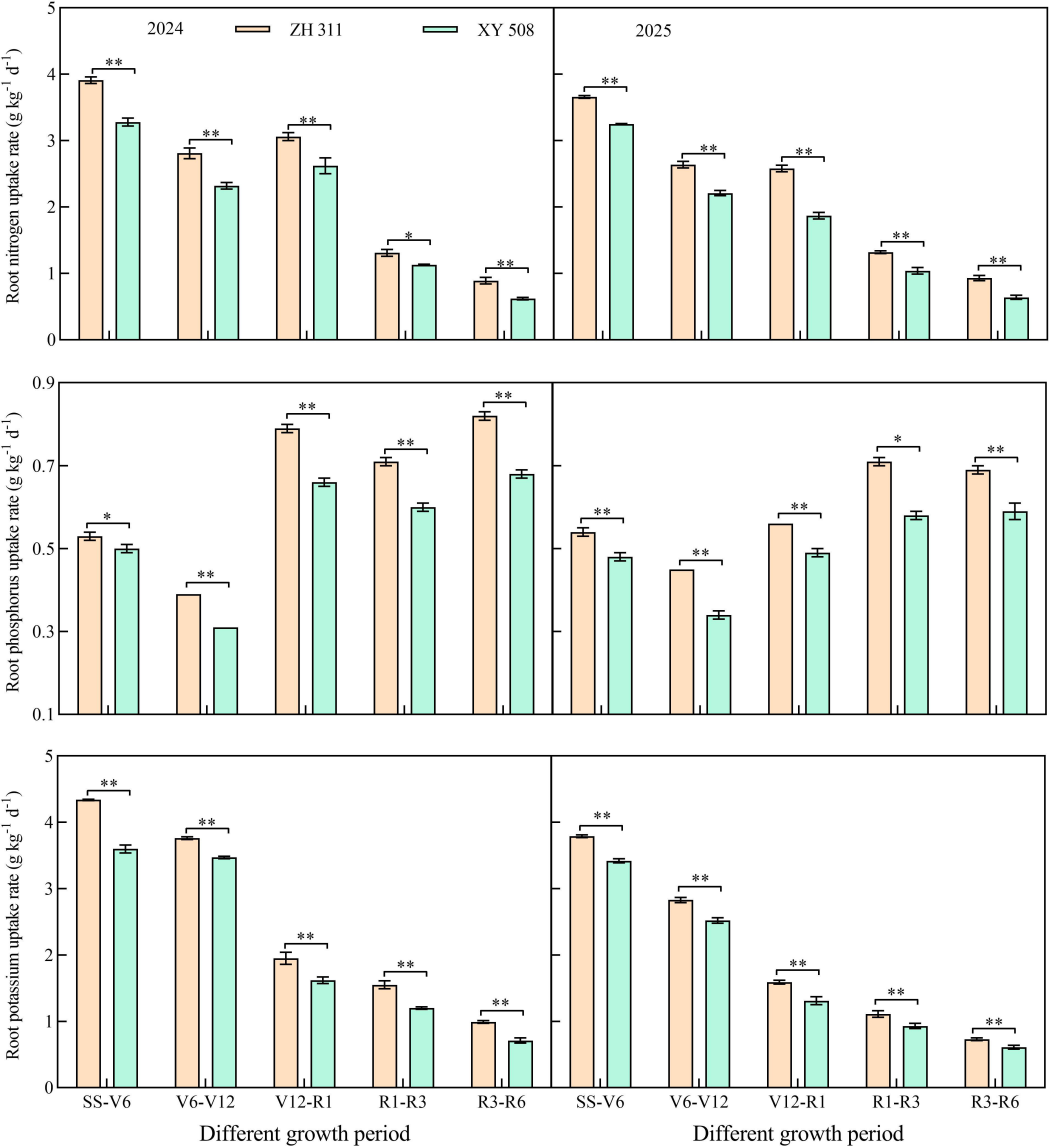
Root N, P, K uptake rate during different growth period.

The N uptake rate of maize roots decreased with growth. In both years, that of the two varieties was the highest in the SS-V6 stage and lowest in the R3-R6 stage (**Figure 3**, **Supplementary Table 2**). The P uptake rate of maize roots in the V12-R1, R1-R3 and R3-R6 stages was significantly higher than that in the other stages. The change in the K uptake rate of maize roots was similar to that of N. That of the two varieties was the highest in the SS-V6 stage and the lowest in the R3-R6 stage over the two years. The absorption rates of N, P, and K in the roots of maize varieties with different N efficiencies differed significantly (*p* < 0.05), and the root N, P, and K absorption rates of ZH 311 were significantly higher than those of XY 508 at all stages. In 2024, the root N absorption rates of ZH 311 in SS-V6, V6-V12, V12-R1, R1-R3 and R3-R6 stages were 19.34%, 21.46%, 16.55%, 16.18%, and 43.78% higher, respectively, and the root P absorption rates were 6.98%, 27.94%, 18.49%, 18.67%, and 20.68% higher than those of XY 508, respectively. The K absorption rates of the roots were 20.61%, 8.19%, 20.15%, 28.94%, and 39.84% higher than those of the roots, respectively. In 2025, root N uptake rates increased by 12.61%, 19.28%, 38.13%, 26.47%, and 45.18%, respectively. The P-uptake rates of the roots were 10.69%, 33.97%, 13.89%, 21.62%, and 17.15% higher, respectively. The K absorption rates of the roots were 10.84%, 12.25%, 21.45%, 19.56%, and 19.94% higher, respectively. The increased rate of root N, P and K uptake rate was the highest in R3-R6, V6-V12, and in the late growth stage (V12-R6), respectively. These results show that ZH 311 had root N, P, and K absorption advantage at each growth stage compared to XY 508, but there were obvious stage differences.

### 3.4 Differences in absorption and utilization efficiency of N, P and K in different N-efficiency maize varieties

The variety and N application had significant effects on the absorption efficiencies of N, P, and K in maize (**Table 5**). The absorption efficiencies of N, P, and K in ZH 311 were significantly higher than those in XY 508 in both years (*p* < 0.01). The average absorption efficiencies of N, P, and K within ZH 311 in 2024 were 21.43%, 25.00%, and 22.32% higher than those in XY 508, respectively, and 25.00%, 22.22%, and 14.58% higher in 2025, respectively, indicating that ZH 311 has a stronger absorption capacity for N, P, and K in the root system than XY 508 and a higher absorption efficiency for N, P, and K. N application significantly affected the absorption efficiency of N, P, and K in maize. The absorption efficiency of N decreased significantly, whereas that of P and K increased significantly. In 2024, compared to N120, the N uptake efficiency of ZH 311 in N240 and N360 decreased by 48.19% and 141.18%, respectively, and that of XY 508 decreased by 63.49% and 128.89%, respectively. By 2025, these were reduced by 53.25%, 156.52%, 53.45%, and 102.27%, respectively. Compared with N0, in 2024, the P absorption efficiency of ZH 311 under N120, N240, and N360 increased by 11.36%, 25.00%, and 18.18%, respectively; the K absorption efficiency increased by 21.62%, 41.44%, and 32.43%, respectively; and that of XY 508 increased by 8.57%, 17.14%, 25.71%, 22.47%, 35.96%, and 44.94%, respectively. In 2025, the P and K uptake efficiencies of ZH 311 increased by 7.50%, 20.00%, 10.00% and 32.93%, 62.60%, 42.68% respectively; whereas XY 508 increased by 9.38%, 15.63%, 25.00% and 32.86%, 52.86%, 64.29%, respectively. The decrease in the N uptake efficiency of ZH 311 was lower than that of XY 508, whereas the increase in the P and K uptake efficiency was higher than that of XY 508, indicating that the N, P, and K uptake efficiencies of the N-efficient variety ZH 311 were higher than the N-inefficient variety XY 508; however, the advantage was more obvious at low and medium N levels.

**Table 5:** Difference of N, P, K absorption efficiency (kg kg^−1^).

Varieties	Nitrogen rate	N		P		K	
		2024	2025	2024	2025	2024	2025
ZH 311	N 0	—	—	0.44 ± 0.01 d	0.40 ± 0.01 d	1.11 ± 0.02 f	0.82 ± 0.02 e
	N 120	1.23 ± 0.02 a	1.18 ± 0.02 a	0.49 ± 0.02 c	0.43 ± 0.01 c	1.35 ± 0.02 c	1.09 ± 0.00 c
	N 240	0.83 ± 0.00 c	0.77 ± 0.01 c	0.55 ± 0.01 a	0.48 ± 0.01 a	1.57 ± 0.02 a	1.33 ± 0.02 a
	N 360	0.51 ± 0.00 e	0.46 ± 0.01 e	0.52 ± 0.01 b	0.44 ± 0.00 b	1.47 ± 0.02 b	1.17 ± 0.02 b
	Mean	**0.85 A**	**0.80 A**	**0.50 A**	**0.44 A**	**1.37 A**	**1.10 A**
XY 508	N 0	—	—	0.35 ± 0.01 g	0.32 ± 0.00 g	0.89 ± 0.01 g	0.70 ± 0.02 f
	N 120	1.03 ± 0.04 b	0.89 ± 0.01 b	0.38 ± 0.01 f	0.35 ± 0.00 f	1.09 ± 0.01 f	0.93 ± 0.02 d
	N 240	0.63 ± 0.01 d	0.58 ± 0.01 d	0.41 ± 0.01 e	0.37 ± 0.00 e	1.21 ± 0.02 e	1.07 ± 0.02 c
	N 360	0.45 ± 0.01 f	0.44 ± 0.01 e	0.44 ± 0.01 d	0.40 ± 0.00 d	1.29 ± 0.02 d	1.15 ± 0.01 b
	Mean	**0.70 B**	**0.64 B**	**0.40 B**	**0.36 B**	**1.12 B**	**0.96 B**
*F* value	Years/Y	101.39**		321.40**		1532.20**	
	Variety/V	798.20**		1135.13**		1272.63**	
	Nitrogen/N	4007.14**		182.45**		1201.81**	
	Y × V	1.62^ns^		18.73**		106.76**	
	Y × N	9.81**		3.89*		2.90^ns^	
	V × N	120.31**		17.10**		61.09**	
	Y × V × N	11.76**		2.10^ns^		1.69^ns^	

The bolded parts are all "Mean", and in the chart, they represent the average values of different varieties and different years (N0, N120, N240, N360) of data.

Values are means ± SD (n = 3). Different lowercase letters in the same column represent a significant difference (p < 0.05); ** indicates the difference was extremely significant at the 1% level (p < 0.01); * indicates the difference was significant at the 5% level (p < 0.05); ns indicates that the difference or relationship being tested is not statistically significant.

The variety type and N application had significant effects on the N, P, and K use efficiencies of maize (**Table 6**). The utilization efficiencies of N, P, and K in ZH 311 were significantly higher than in XY 508 (*p* < 0.01). On average, the utilization efficiencies of N, P, and K in ZH311 in 2024 were 12.81%, 33.08%, and 13.59% higher than those of XY 508, respectively, and 16.44%, 42.21%, and 9.65% higher in 2025, respectively, indicating that ZH 311 has a stronger utilization ability and higher utilization efficiency than XY 508. N application had a significant effect on the utilization efficiency of N, P, and K in maize; however, there were obvious differences among the varieties. The N, P, and K use efficiency of ZH 311 was significantly lower than that of N240 at the N120 and N360 levels in both years (*p* < 0.05), among which the N use efficiency was the lowest at the N360 level, and the P and K use efficiencies were the lowest at the N120 level. The N utilization efficiency of XY 508 decreased significantly with an increase in the N level over two years, and the utilization efficiency of P and K increased significantly. The N utilization efficiency was the lowest at the N360 level, and the P and K utilization efficiencies were the lowest under N120 level. These results showed that the N, P and K utilization efficiencies of ZH 311 were obviously higher than those of XY 508, but the advantages were more obvious under medium and low N levels, while the advantages decreased under the high N treatment.

**Table 6:** Difference of N, P, K apparent utilization (%).

Varieties	Nitrogen rate	N		P		K	
		2024	2025	2024	2025	2024	2025
ZH 311	N 0	—	—	—	—	—	—
	N 120	34.91 ± 0.32 b	33.22 ± 0.97 b	5.63 ± 0.53 d	5.01 ± 0.30 d	25.97 ± 0.62 e	26.93 ± 1.27 d
	N 240	37.85 ± 0.47 a	36.34 ± 0.76 a	11.36 ± 1.04 a	9.33 ± 0.32 a	47.59 ± 0.14 a	47.12 ± 0.34 a
	N 360	21.04 ± 0.15 e	24.59 ± 0.76 e	8.83 ± 0.13 b	8.09 ± 0.29 b	36.00 ± 0.44 c	36.42 ± 0.59 c
	Mean	**31.27 A**	**31.38 A**	**8.61 A**	**7.48 A**	**36.52 A**	**36.82 A**
XY 508	N 0	—	—	—	—	—	—
	N 120	33.57 ± 0.49 c	29.80 ± 0.65 c	3.91 ± 0.28 e	3.08 ± 0.13 e	20.72 ± 0.61 f	21.74 ± 0.69 e
	N 240	28.84 ± 0.43 d	27.19 ± 0.79 d	7.18 ± 0.43 c	5.21 ± 0.44 d	34.52 ± 1.11 d	37.18 ± 0.49 c
	N 360	20.74 ± 0.44 e	23.84 ± 0.56 e	8.32 ± 0.33 b	7.49 ± 0.36 c	41.19 ± 0.86 b	41.80 ± 0.55 b
	Mean	**27.72 B**	**26.95 B**	**6.47 B**	**5.26 B**	**32.15 B**	**33.58 B**
*F* value	Years/Y	2.70^ns^		61.71**		16.22**	
	Variety/V	399.95**		213.47**		312.30**	
	Nitrogen/N	1151.92**		292.72**		2622.49**	
	Y × V	4.92*		0.07^ns^		6.81*	
	Y × N	86.68**		7.89**		0.67^ns^	
	V × N	169.04**		49.82**		516.11**	
	Y × V × N	2.25^ns^		0.06^ns^		5.43*	

The bolded parts are all "Mean", and in the chart, they represent the average values of different varieties and different years (N0, N120, N240, N360) of data.

Values are means ± SD (n = 3). Different lowercase letters in the same column represent a significant difference (p < 0.05); ** indicates the difference was extremely significant at the 1% level (p < 0.01); * indicates the difference was significant at the 5% level (p < 0.05); ns indicates that the difference or relationship being tested is not statistically significant.

**Figure 4:**
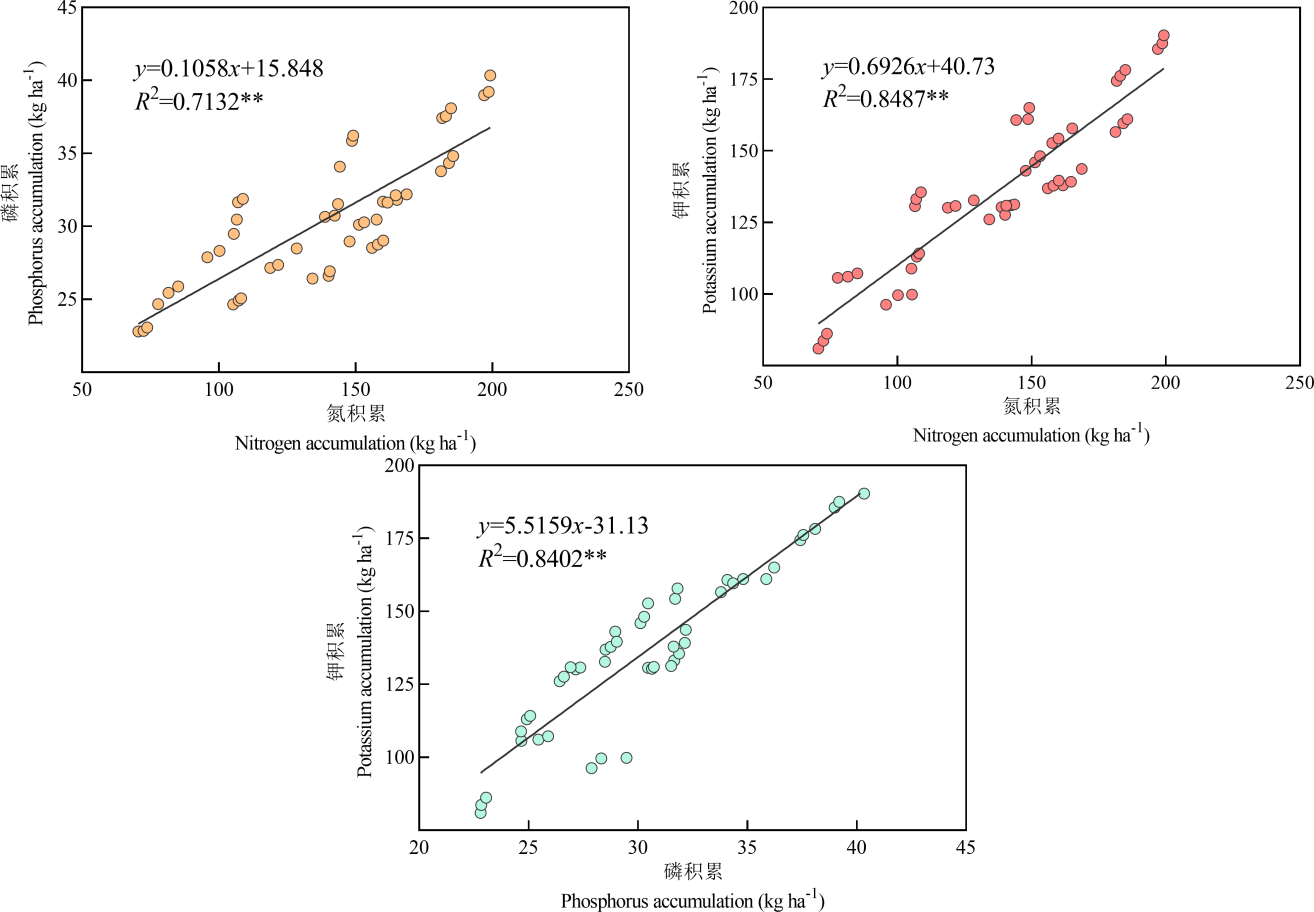
Correlation analysis of N, P and K accumulation in maize.

Linear regression analysis was performed to investigate the relationships between N, P, and K accumulation in maize varieties ZH 311 and XY 508 (**Figure 4**). Strong positive correlations were observed between the accumulation of N and P (*R*^2^ = 0.7132), N and K (*R*^2^ = 0.8487), and P and K (*R*^2^ = 0.8402**). These results demonstrate a synergistic relationship in the uptake and accumulation of N, P, and K in maize.

**Figure 5:**
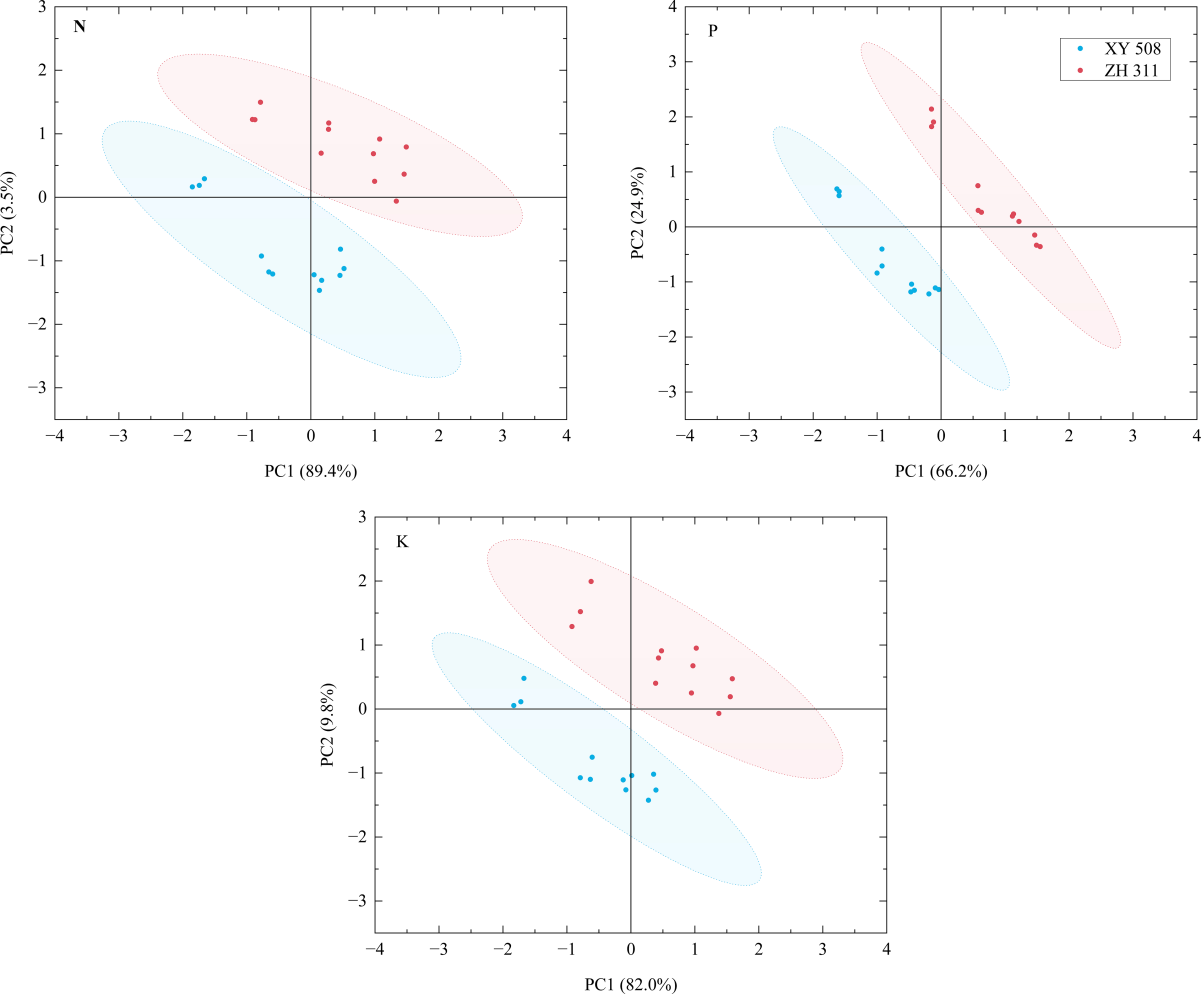
PCA of N, P, and K absorption and utilization in ZH 311 and XY 508. PC1 and PC2 explained 57.54% and 32.45% of the total variation, respectively. The loading coefficients of the nutrient absorption and apparent utilization efficiency indicators are shown in **Table 7**.

Principal component analysis (PCA) showed that the two maize varieties were separated based on N, P, and K absorption and utilization characteristics (**Figure 5**). PC1 and PC2 explained 57.54% and 32.45% of the total variation, respectively. The loading matrix further indicated that P absorption efficiency, K absorption efficiency, P apparent utilization efficiency, and K apparent utilization efficiency contributed strongly to PC1, whereas N absorption efficiency and N apparent utilization efficiency contributed mainly to PC2 (**Table 7**).

**Table 7:** PCA loading matrix of nutrient absorption and apparent utilization efficiency indicators.

Indicator	PC1	PC2	PC3
N absorption efficiency	-0.450	-0.879	0.079
P absorption efficiency	0.839	-0.451	0.284
K absorption efficiency	0.898	-0.330	0.247
N apparent utilization efficiency	-0.079	-0.902	-0.413
P apparent utilization efficiency	0.986	0.014	-0.098
K apparent utilization efficiency	0.872	0.222	-0.413
Contribution rate (%)	57.54	32.45	8.31
Cumulative contribution (%)	57.54	89.99	98.30

PCA was conducted using six standardized nutrient-efficiency indicators, including N, P, and K absorption efficiencies and N, P, and K apparent utilization efficiencies. PC1, PC2, and PC3 explained 57.54%, 32.45%, and 8.31% of the total variation, respectively. Values are loading coefficients; larger absolute values indicate greater contributions to the corresponding principal component.

### 3.5 Yield response and yield components of maize varieties with different N efficiency

Nitrogen application significantly affected maize yield and yield components in both years (**Table 8**). ZH 311 achieved the highest grain yield under N240 in both years, indicating that moderate N input was sufficient to support high yield in the N-efficient variety.

**Table 8:** Yield components and grain yield of maize varieties under different N rates.

Year	Variety	N rate	Ear rows	Kernels per row	Kernels per ear	100-kernel weight (g)	Grain yield (t ha^−1^)
2024	ZH 311	N0	16.14 ± 0.20	23.86 ± 0.81	366.66 ± 14.27	27.19 ± 0.14	5.94 ± 0.14
2024	ZH 311	N120	17.05 ± 0.24	26.97 ± 0.19	437.96 ± 3.53	28.34 ± 0.27	7.24 ± 0.10
2024	ZH 311	N240	17.71 ± 0.33	27.77 ± 0.07	468.39 ± 7.62	29.17 ± 0.04	8.24 ± 0.03
2024	ZH 311	N360	17.48 ± 0.12	27.53 ± 0.27	458.34 ± 2.93	28.50 ± 0.07	7.97 ± 0.16
2024	XY 508	N0	14.06 ± 0.10	21.17 ± 0.74	283.47 ± 8.56	27.96 ± 0.36	5.20 ± 0.09
2024	XY 508	N120	15.12 ± 0.42	22.61 ± 1.08	325.17 ± 6.77	30.04 ± 0.09	6.10 ± 0.08
2024	XY 508	N240	15.89 ± 0.32	25.05 ± 0.17	379.06 ± 7.89	31.02 ± 0.25	7.11 ± 0.10
2024	XY 508	N360	16.41 ± 0.15	25.62 ± 0.15	400.41 ± 4.86	31.66 ± 0.10	7.46 ± 0.07
2025	ZH 311	N0	14.98 ± 0.32	23.69 ± 0.93	337.96 ± 13.67	26.67 ± 0.24	5.48 ± 0.14
2025	ZH 311	N120	16.50 ± 0.52	26.17 ± 0.32	411.18 ± 8.12	28.01 ± 0.09	6.55 ± 0.08
2025	ZH 311	N240	17.51 ± 0.07	26.88 ± 1.11	448.11 ± 16.91	29.08 ± 0.01	7.46 ± 0.21
2025	ZH 311	N360	17.14 ± 0.48	26.45 ± 0.22	431.63 ± 10.17	28.46 ± 0.01	6.75 ± 0.24
2025	XY 508	N0	14.13 ± 0.31	20.88 ± 0.47	281.07 ± 11.40	27.72 ± 0.20	4.64 ± 0.06
2025	XY 508	N120	15.10 ± 0.15	23.54 ± 0.70	338.60 ± 7.06	29.18 ± 0.04	5.71 ± 0.10
2025	XY 508	N240	15.46 ± 0.23	24.56 ± 0.41	361.58 ± 1.23	29.95 ± 0.02	6.25 ± 0.08
2025	XY 508	N360	15.58 ± 0.33	25.40 ± 0.18	377.02 ± 8.10	30.36 ± 0.05	6.47 ± 0.03

Values are means ± SD (n = 3). Grain yield was converted from kg mu−1 to t ha−1 using the conversion factor 0.015.

For XY 508, grain yield increased with increasing N application and reached the highest value under N360 in both years, suggesting that the N-inefficient variety was more dependent on higher N input to obtain higher yield.

Across the same N levels, ZH 311 consistently produced higher grain yield than XY 508. The yield advantage of ZH 311 was more evident under low and medium N levels, especially under N120 and N240, whereas the advantage decreased under N360. 

## 4 Discussion

### 4.1 Synergistic absorption of N, P and K by maize roots

There were obvious genotypic differences in the root phenotypes of the crops, and the absorption capacities of N, P, K, and water also differed (Schmidt et al., 2022). Cheng et al. (2017) found that the characteristics of the root tip are the main factors affecting N uptake, while Li et al. (2022a) showed that root development is closely related to nutrient uptake in maize varieties with different N efficiencies. In the present study, ZH 311 maintained higher root dry matter than XY 508 at the later growth stage, especially at R6, which may have supported its higher N, P, and K accumulation and absorption rates. This indicates that the nutrient-efficiency advantage of ZH 311 was related not only to N uptake but also to a stronger root capacity for acquiring P and K.

The absorption of N, P, and K by plant roots has a synergistic effect; however, the carriers of N, P, and K in the cells differ significantly. The different accumulation peaks observed in this study further indicate that N, P, and K coordination was stage-specific rather than completely synchronous. N accumulation peaked during V12-R1, P accumulation was more active during the later reproductive stage, and K accumulation peaked earlier during V6-V12. Therefore, the positive correlations among N, P, and K accumulation indicate coordinated nutrient acquisition, but this coordination occurred through different growth-stage demands.

### 4.2 Synergistic utilization of N, P and K in maize

N, P, and K are the most important nutrients for plants, and their absorption and utilization rates reflect plant yield (Kumari et al., 2022; Sun et al., 2021; Zhu et al., 2019). The absorption, assimilation, and transport of N, P, and K have clear interactions in plants, and an appropriate nutrient supply is important for improving nutrient-use efficiency. In this study, the higher absorption and apparent utilization efficiencies of ZH 311 indicated that the N-efficient variety had a stronger capacity to convert nutrient uptake into root and plant nutrient accumulation, whereas XY 508 depended more strongly on increased fertilizer supply to improve nutrient accumulation.

PCA showed that the principal component space of the N absorption and utilization characteristics was different for ZH 311 and XY 508. The loading matrix further showed that PC1 was mainly associated with P and K absorption and apparent utilization efficiencies, whereas PC2 was mainly associated with N absorption efficiency and N apparent utilization efficiency. This indicates that varietal differences were not limited to N uptake and utilization, but also involved P and K efficiency.

The results of this study showed that the synergistic absorption and utilization of N, P, and K differed between the two maize varieties. The newly added yield results further supported this nutrient-efficiency advantage. ZH 311 achieved its highest yield under N240 in both years, whereas XY 508 reached the highest yield under N360, suggesting that the N-efficient variety could obtain high yield under moderate N input, while the N-inefficient variety required a higher N rate to approach its yield potential.

In this study, we clarified the synergistic absorption and utilization effects of N, P, and K in maize, but we were unable to determine the detailed mechanism involved in their synergistic absorption and utilization effects. In addition, the decline in the efficiency advantage of ZH 311 under N360 suggests that excessive N application may exceed the efficient utilization threshold of the N-efficient genotype, reducing the marginal benefit of additional fertilizer.

Only the basic nutrient status of the 0–30 cm soil layer was measured, and root exudates, rhizosphere nutrient activation, and transporter expression were not directly determined. Therefore, the roles of NRT, PHT, and K^+^ transporter systems in N-P-K coordination should be further verified in future studies.

## 5 Conclusions

The absorption efficiency and accumulation of N, P, and K in the roots of ZH 311 were significantly higher than those of XY 508, and there were significant positive correlations between N-P, N-K, and P-K. Further analysis revealed the absorption rates of N and P were the highest and lowest, respectively. Accumulation of N, P, and K was the highest in the V12-R1, R3-R6, and V6-V12 stages, respectively. These results indicate stage-specific coordination of N, P, and K uptake rather than completely synchronous uptake. PCA further showed that P and K absorption and utilization efficiencies, together with N absorption and apparent utilization efficiencies, were important indicators distinguishing the two varieties. These results show the superior N, P, and K uptake and utilization efficiency of N-efficient varieties compared to N-inefficient ones. Yield results indicated that ZH 311 achieved the highest yield under N240, whereas XY 508 reached the highest yield under N360, suggesting that selecting N-efficient maize varieties with stronger coordinated P and K uptake capacity and applying a moderate N rate may improve nutrient-use efficiency and support high-yield maize production.

## Data Availability

The original contributions presented in the study are included in the article/supplementary material. Further inquiries can be directed to the corresponding author.
