# Supplementary material for: Synergistic nutrient uptake and utilization in maize varieties with different nitrogen efficiencies
Source: Front Plant Sci. 2026 Jul 29;17:1867272. doi: 10.3389/fpls.2026.1867272 (PMC13462741; doi:10.3389/fpls.2026.1867272)
Supplement: Supplementary file 1 [file Table1.docx]

|  | **Stage** | **SS-V6** | | | **V6-V12** | | | **V12-R1** | | | **R1-R3** | | | **R3-R6** | | |
| --- | --- | --- | --- | --- | --- | --- | --- | --- | --- | --- | --- | --- | --- | --- | --- | --- |
|  |  | **N** | **P** | **K** | **N** | **P** | **K** | **N** | **P** | **K** | **N** | **P** | **K** | **N** | **P** | **K** |
| *F* value | Years/Y | 31.60** | 124.98** | 4.56* | 136.47** | 5.45* | 360.33** | 60.53** | 929.56** | 32.15** | 1.01^ns^ | 20.68** | 119.06** | 4.01^ns^ | 119.98** | 90.49** |
|  | Cultivar/C | 190.46** | 130.77** | 176.81** | 111.62** | 302.68** | 37.31** | 76.80** | 103.36** | 46.43** | 79.93** | 194.47** | 97.23** | 281.60** | 238.38** | 106.55** |
|  | Nitrogen/N | 661.08** | 740.71** | 256.26** | 271.91** | 82.09** | 75.72** | 121.60** | 10.19** | 49.75** | 161.20** | 65.00** | 57.03** | 133.13** | 13.28** | 62.04** |
|  | Y × C | 11.97** | 1.70^ns^ | 26.23** | 2.39^ns^ | 7.43* | 0.01^ns^ | 2.97^ns^ | 38.27** | 1.55^ns^ | 0.15^ns^ | 4.80* | 16.33** | 0.29^ns^ | 2.55^ns^ | 13.36** |
|  | Y × N | 1.89^ns^ | 13.79** | 1.78^ns^ | 6.80** | 10.11** | 4,00* | 0.50^ns^ | 2.99* | 0.48^ns^ | 17.38** | 4.68** | 1.05^ns^ | 7.42** | 3.42* | 0.12^ns^ |
|  | C × N | 22.32** | 17.85** | 7.81** | 9.48** | 1.73^ns^ | 4.44* | 1.89^ns^ | 2.71^ns^ | 5.33** | 7.66** | 11.54** | 2.70^ns^ | 15.08** | 3.93* | 3.57* |
|  | Y × C × N | 2.94* | 6.79** | 2.54^ns^ | 3.66* | 1.62^ns^ | 1.80^ns^ | 1.13^ns^ | 3.56* | 0.87^ns^ | 7.55** | 8.34** | 0,90^ns^ | 9.05** | 0.87^ns^ | 0.33^ns^ |

Supplementary table 1. Analysis of variance of N, P, K accumulation during different growth period

Supplementary table 2 Variance analysis of root N, P, K absorption rate during different growth period

|  | **Stage** | **SS-V6** | | | **V6-V12** | | | **V12-R1** | | | **R1-R3** | | | **R3-R6** | | |
| --- | --- | --- | --- | --- | --- | --- | --- | --- | --- | --- | --- | --- | --- | --- | --- | --- |
|  |  | **N** | **P** | **K** | **N** | **P** | **K** | **N** | **P** | **K** | **N** | **P** | **K** | **N** | **P** | **K** |
| *F* value | Years/Y | 1033.76** | 665.04** | 135.90** | 1244.99** | 34.35** | 240.09** | 21518.26** | 285.02** | 19188.76** | 75.18** | 1106.45** | 1284.89** | 537.31** | 222.12** | 427.32** |
|  | Cultivar/C | 310.35** | 65.52** | 279.29** | 44.82** | 123.37** | 159.36** | 335.60** | 95.65** | 265.91** | 591.99** | 136.33** | 73.22** | 280.95** | 104.57** | 79.48** |
|  | Nitrogen/N | 159.74** | 9.26** | 6.21** | 23.89** | 46.19** | 3.33* | 427.14** | 5.88** | 73.08** | 250.30** | 7.78** | 14.33** | 186.47** | 4.39* | 58.75** |
|  | Y × C | 4.49* | 8.70** | 55.84** | 7.47** | 5.24* | 0.13^ns^ | 14.44** | 9.03** | 5.10** | 37.16** | 47.95** | 0.16^ns^ | 0.14^ns^ | 2.66^ns^ | 3.11^ns^ |
|  | Y × N | 16.16** | 36.80** | 65.83** | 7.48** | 27.87** | 2.75^ns^ | 109.03** | 10.96* | 16.59** | 12.91** | 15.59** | 3.08* | 52.41** | 2.92* | 2.49^ns^ |
|  | C × N | 25.90** | 1.01^ns^ | 1.52^ns^ | 0.62^ns^ | 0.60^ns^ | 0.83^ns^ | 17.84** | 3.72* | 4.63** | 12.50** | 1.44^ns^ | 3.34* | 23.48** | 1.22^ns^ | 0.99^ns^ |
|  | Y × C × N | 5.64** | 0.66^ns^ | 1.41^ns^ | 1.37^ns^ | 7.15** | 0.05^ns^ | 8.52** | 1.40^ns^ | 8.60** | 0.67^ns^ | 0.84^ns^ | 1.86^ns^ | 0.43^ns^ | 0.17^ns^ | 0.20^ns^ |
